# Identification of Phosphohistone H3 Cutoff Values Corresponding to Original WHO Grades but Distinguishable in Well-Differentiated Gastrointestinal Neuroendocrine Tumors

**DOI:** 10.1155/2018/1013640

**Published:** 2018-03-27

**Authors:** Min Jeong Kim, Mi Jung Kwon, Ho Suk Kang, Kyung Chan Choi, Eun Sook Nam, Seong Jin Cho, Hye-Rim Park, Soo Kee Min, Jinwon Seo, Ji-Young Choe, Hyoung-Chul Park

**Affiliations:** ^1^Department of Surgery, Kangdong Sacred Heart Hospital, Hallym University College of Medicine, 150 Seongan-ro, Gangdong-gu, Seoul 05355, Republic of Korea; ^2^Department of Pathology, Hallym University Sacred Heart Hospital, Hallym University College of Medicine, 170beon-gil, 22 Gwanpyeong-ro, Dongan-gu, Anyang-si, Gyeonggi-do 14068, Republic of Korea; ^3^Research Institute for Complementary & Alternative Medicine, Hallym University, 40 Seokwoo-Dong, Hwaseong, Gyeonggi-do 445-170, Republic of Korea; ^4^Department of Internal Medicine, Hallym University Sacred Heart Hospital, Hallym University College of Medicine, 170beon-gil, 22 Gwanpyeong-ro, Dongan-gu, Anyang-si, Gyeonggi-do 14068, Republic of Korea; ^5^Department of Pathology, Chuncheon Sacred Heart Hospital, Hallym University College of Medicine, Chuncheon, Republic of Korea; ^6^Department of Pathology, Kangdong Sacred Heart Hospital, Hallym University College of Medicine, 150 Seongan-ro, Gangdong-gu, Seoul 05355, Republic of Korea; ^7^Department of Surgery, Hallym University Sacred Heart Hospital, Hallym University College of Medicine, 170beon-gil, 22 Gwanpyeong-ro, Dongan-gu, Anyang-si, Gyeonggi-do 14068, Republic of Korea

## Abstract

Mitotic counts in the World Health Organization (WHO) grading system have narrow cutoff values. True mitotic figures, however, are not always distinguishable from apoptotic bodies and darkly stained nuclei, complicating the ability of the WHO grading system to diagnose well-differentiated neuroendocrine tumors (NETs). The mitosis-specific marker phosphohistone H3 (PHH3) can identify true mitoses and grade tumors reliably. The aim of this study was to investigate the correspondence of tumor grades, as determined by PHH3 mitotic index (MI) and mitotic counts according to WHO criteria, and to determine the clinically relevant cutoffs of PHH3 MI in rectal and nonrectal gastrointestinal NETs. Mitotic counts correlated with both the Ki-67 labeling index and PHH3 MI, but the correlation with PHH3 MI was slightly higher. The PHH3 MI cutoff ≥4 correlated most closely with original WHO grades for both rectal NETs. A PHH3 MI cutoff ≥4, which could distinguish between G1 and G2 tumors, was associated with disease-free survival in patients with rectal NETs, whereas that cutoff value showed marginal significance for overall survival in patient with rectal NETs. In conclusion, the use of PHH3 ≥4 correlated most closely with original WHO grades.

## 1. Introduction

**Figure 1:**
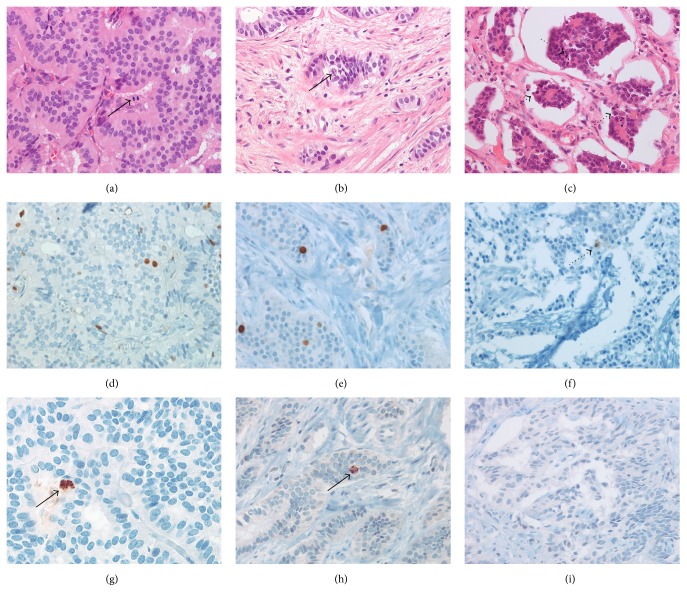
Mitotic figures* (Arrows)* in a rectal neuroendocrine tumor (a, d, g) and a colonic neuroendocrine tumor (b, e, h) stained with H&E (a)–(c), Ki-67 (d)–(f), and PHH3 (g)–(i). (d)-(e) Ki-67 is more frequently positive in tumor cells, whereas (g)-(h) PHH3 highlights mitosis-specific nuclei, aiding in recognition. (c) Apoptotic bodies* (Dotted arrows)* mimicking mitosis are found in gastric neuroendocrine tumors. (f) Faint Ki-67 staining in an apoptotic nucleus, apparently false-positive. (i) Lack of PHH3 staining of apoptotic cells.

Neuroendocrine tumors (NETs) are uncommon, heterogeneous groups of neoplasms, with most (54%) developing in the gastrointestinal tract [1–4]. The incidence and prognosis of gastrointestinal NETs depend on the tumor primary site, with the highest frequencies observed in the rectum (17.7%), small intestine (17.3%), and colon (10.1%), followed by the stomach (6.0%) and appendix (3.1%) and with survival ranging from 6 months to more than 20 years [1–4]. Gastrointestinal NETs largely arise from enterochromaffin and enteroglucagon cells found in the lamina propria and submucosa [5]. Histologically, NETs are composed of an organoid pattern of cells arranged into trabeculae, acini, or solid nests, separated by delicate and vascular stroma, which allows for easy recognition on low-power microscopic examination [5] (Figures 1(a)-1(b)). Well-differentiated NETs, which have malignant potential, are characterized cytologically by bland uniform cells with round to oval nuclei, indistinct nucleoli, and coarsely granular chromatin [6, 7]. Distant metastasis resulting from unexpected tumor aggressiveness is therefore of clinical concern in patients with well-differentiated NETs [8–11].

The most important prognostic indicator in gastrointestinal NETs is the World Health Organization (WHO) grading system, which categorizes gastrointestinal NETs into three grades (G1, G2, and G3), based on mitotic counts and/or Ki-67 labeling index (LI). G1 NETs are low grade tumors, with <2 mitoses/10 high-power fields (HPFs) and/or Ki-67 LI <3%; G2 NETs are intermediate grade tumors (2–20 mitoses/10 HPFs and/or Ki-67 LI 3%–20%), and G3 NETs are high grade tumors (>20 mitoses/10 HPFs and/or Ki-67 LI >20%) [12, 13]. Most gastrointestinal NETs are G1 (59.7%) and G2 (31.2%), with few (9.1%) classified as G3 [4]. Because true mitotic figures are sometimes indistinguishable from darkly stained and/or shrunken irregular nuclei, apoptotic bodies, and karyorrhectic debris on hematoxylin and eosin (H&E) staining, identification of true mitotic figures is not always straightforward [5] (Figure 1(c)). Discrepancies have therefore been observed in correlations between Ki-67 and mitotic counts in various tumor types [14–16]. It may be difficult to unequivocally identify a mitotic figure versus apoptotic cells or karyorrhectic cells [16]. Manually calculating Ki-67 LI in 500–2000 cells is highly labor-intensive [14, 17]. The narrow cutoffs in mitotic counts and Ki-67 LI between G1 and G2 well-differentiated NETs may result in false upgrading or downgrading of tumors. Therefore, the supportive method for counting mitotic figures and Ki-67 LI is necessary to confirm the limitation of the current criteria for precisely determining the prognosis of patients with gastrointestinal well-differentiated NETs [14, 17].

Phosphohistone H3 (PHH3), a core histone protein reaching a maximum during mitosis, is a mitosis-specific marker, making it useful in counting mitotic figures and for mitotic grading. PHH3 facilitates the counting of mitoses and can be used to predict prognosis in patients with several types of gastrointestinal neoplasm, including pancreatic NETs [14, 18–20]. However, the ability of PHH3 mitotic index (MI) to grade gastrointestinal NETs, especially for differentiating between G1 and G2 well-differentiated NETs, has not yet been fully evaluated. Furthermore, the clinically relevant cutoffs for PHH3 MI in rectal and nonrectal NETs have not yet been determined.

The aim of this study was to compare tumor grades determined using the PHH3 MI and those determined by mitotic counts according to WHO criteria and to determine the clinically relevant cutoffs of PHH3 MI. In this study, Ki-67 LI was calculated digitally, because manual calculation may be a confounding factor.

## 2. Materials and Methods

### 2.1. Patients and Histologic Evaluation

This study retrospectively evaluated 141 patients with primary gastrointestinal NETs who underwent endoscopic or surgical resection at Hallym University Sacred Heart Hospital between 2005 and 2015. Only patients diagnosed with primary gastrointestinal NETs, who had not been treated with chemotherapy or targeted drug therapy at the time of tumor excision and whose formalin-fixed, paraffin-embedded (FFPE) tumor tissue blocks were available for analysis, were included in this study. The medical records of each patient were reviewed, and their demographic information, radiological data, treatment details, tumor recurrence, and survival status were recorded. All H&E-stained slides were reviewed by a gastrointestinal pathologist (MJK) to confirm the diagnosis and to reevaluate histopathological characteristics, including tumor size, mitotic count, tumor grade, resection margins, depth of invasion, lymphatic invasion, venous invasion, and perineural invasion. Staging was based on the 8th edition of American Joint Committee on Cancer staging system. The study was approved by the Institutional Review Board of the Hallym University Sacred Heart Hospital.

### 2.2. Immunohistochemistry

Immunohistochemical staining was performed on 4 *μ*m thick FFPE tumor tissue sections using the BenchMark XT automated tissue staining system (Ventana Medical Systems, Inc., Tucson, AZ, USA), according to the manufacturer's instructions, as described in [21–24]. The primary antibodies were directed against PHH3 (polyclonal, 1 : 100; Cell Marque, Rocklin, CA, USA) and Ki-67 (1 : 250, clone MIB-1, Dako). Slides were incubated with primary antibody 37°C for 40 min, washed, and incubated with a secondary antibody (universal horseradish peroxidase (HRP) Multimer; Ventana Medical Systems) for 8 min at 37°C. After washing, the tissue sections were incubated with a chromogen diaminobenzidine (ultraView Universal DAB Kit, Ventana Medical Systems) and counterstained with hematoxylin.

### 2.3. Slide Scoring

Mitotic counts on both H&E- and PHH3-stained slides were counted in 50 high-powered fields (HPFs; 40 × objective, 10 × eyepiece with a field diameter of 0.55 mm and an area of 0.237 mm^2^; Olympus microscope BX51, Tokyo, Japan). PHH3 MI was calculated from the mean mitotic count (mean number of mitoses/10 HPFs) and the mean numbers of PHH3-positive nuclei/10 HPFs were calculated as the number of mitoses/10 HPFs and the number of PHH3-positive nuclei/10 HPFs to attain the PHH3 MI, respectively [14, 18, 25]. Mitotic figures were considered as cells in metaphase (clumped chromatin and chromatin arranged in a plane) and anaphase/telophase (separated clumped chromatin), as previously described [14]. Hyperchromatic or pyknotic nuclei were not counted, because these cells could represent cells undergoing necrosis or apoptosis, as previously described [14].

Ki-67 LI was assessed using a GenASIs capture and analysis system (Applied Spectral Imaging, Carlsbad, CA, USA). Briefly, the highest labeled region at low magnification was selected, and the area was viewed at ×200 magnification. These captured images were analyzed with GenASIs software to quantify the positive tumor cells in each tumor region. Ki-67-positive lymphocytes were manually removed. At least 500 tumor cells per sample were counted to determine the percentage of cells that were positive for Ki-67, and Ki-67 LI was automatically calculated.

Grades of H&E- and anti-PHH3-stained sections were determined independently. Tumors were classified as G1 (<2 mitoses per 10 HPFs and/or Ki-67 LI <3%), G2 (2–20 mitoses per 10 HPFs and/or Ki-67 LI 3%–20%), and G3 or NEC (>20 mitoses per HPF or Ki-67 >20%), according to the WHO 2010 classification [12, 13].

### 2.4. Statistical Analyses

Categorical variables were compared using Pearson's chi-squared test or two-tailed Fisher's exact test, and continuous variables, which were presented as means ± SD, were compared using Student's *t*-test. The Spearman rank correlation test was used to assess the relationships between mitotic counts, Ki-67 LI, and PHH3 mitotic index. The results obtained with the WHO grading system with those derived from PHH3-applied modified grading were compared by assessing the concordance rate (number of samples in which the two methods agreed/number of total samples) with the kappa (*κ*) statistic. Concordance rate was defined as the proportion of similar results achieved using 2 different methods, among total number of cases. The kappa value was evaluated to measure the degree of agreement between 2 different grading methods. Kappa values ≤0.20, 0.21–0.40, 0.41–0.60, 0.61–0.80, and ≥0.81 were regarded as indicating slight, fair, moderate, substantial, and almost perfect agreement, respectively. The volume under the receiver operator characteristic (ROC) curve was drawn to determine the optimal cutoff value in terms of sensitivity and specificity for WHO grades 1 and 2 or 3 by PHH3 MI.

Overall survival was defined as the time from the date of initial surgery until death or the end of the stay (May 2017). Disease-free survival was defined as the time from the date of initial surgery until a documented relapse, including locoregional recurrence and distant metastasis, or the end of the study. Survival parameters were calculated using the Kaplan-Meier method and compared by log-rank tests. All statistical analyses were performed using SPSS software (version 18; SPSS Inc., Chicago, IL, USA), with *P* values <0.05 considered statistically significant.

## 3. Results

### 3.1. Patient and Tumor Characteristics

Table 1 summarizes the characteristics of patients with rectal and nonrectal NETs. The study enrolled 141 patients, 88 men and 53 women, of median age 49 years (range 10–80 years). Of these patients, 115 (81.6%) had rectal NETs and 26 (18.4%) had nonrectal NETs. The nonrectal NETs included 12 (8.5%) originating from the stomach, eight (5.7%) from the appendix, three (2.1%) from the duodenum, and three (2.1%) from the colon. Tumor tissue was obtained by endoscopic resection from 112 (89.6%) patients with rectal NETs and 13 (10.4%) with nonrectal NETs. The remaining three rectal and 13 nonrectal NETs were resected surgically. Mean tumor size was 0.65 cm (range, 0.1–3.5 cm). Resection margins of 22 (15.6%) tumors were positive. Thirteen patients experienced recurrences and eight died during the follow-up period.

Several demographic and clinical characteristics differed significantly in patients with rectal and nonrectal NETs. Patients with rectal NETs were significantly younger in age (48 versus 56 years, *P* = 0.001) and had smaller-sized tumors (0.58 ± 0.35 versus 0.92 ± 0.90 cm, *P* < 0.001). The depth of tumor invasion was more superficial in patients with rectal NETs, with 99.1% of these patients having tumors confined to the submucosa, whereas a higher percentage of nonrectal NETs (19.2%) infiltrated the muscle layer or adipose tissue (*P* = 0.001). Tumor stage (*P* = 0.007) and tumor grade (*P* = 0.001) were significantly lower in patients with rectal than nonrectal NETs, with 83.5% and 58.3%, respectively, having grade 1 tumors. Recurrence (5.2% versus 26.9%,  *P* = 0.001) and mortality (3.5% versus 15.4%,  *P* = 0.018) rates were also significantly lower in patients with rectal than nonrectal NETs.

### 3.2. Mitotic Counts, PHH3, and Ki-67 LI of Rectal and Nonrectal NETs

**Figure 2:**
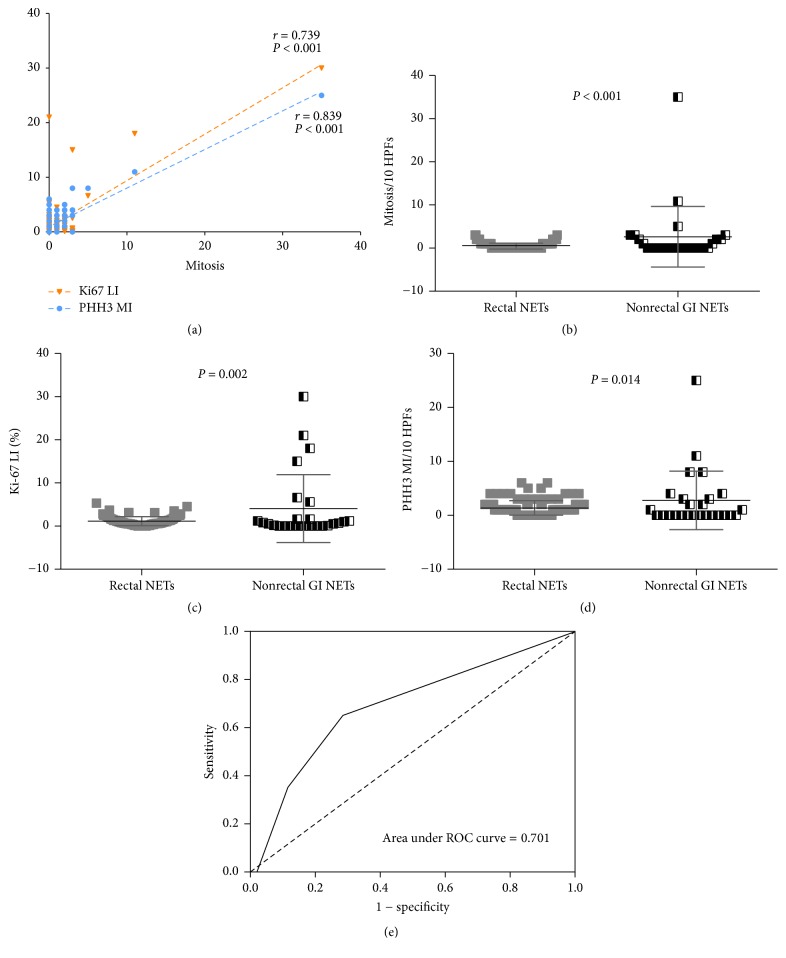
(a) Correlations of mitotic counts obtained from H&E slides with Ki-67 LI and PHH3 MI. Comparisons of mitosis (b), Ki-67 LI (c), and PHH3 MI (d) in rectal and nonrectal neuroendocrine tumors of the gastrointestinal tract. (e) Receiver operating characteristic (ROC) curve for PHH3 MI with original WHO grades 1 and 2.

In all 141 NETs, significant positive correlations were observed between mitotic counts and Ki-67 LI (*r* = 0.739, *P* < 0.001), between mitotic counts and PHH3 MI (*r* = 0.839, *P* < 0.001) (Figure 2(a)), and between PHH3 MI and Ki-67 LI (*r* = 0.724, *P* < 0.001). All of these three parameters, however, differed significantly in rectal and nonrectal NETs. The mean numbers of mitotic counts (0.55 ± 0.79/10 HPFs [range, 0–3/10 HPFs] versus 2.62 ± 7.03/10 HPFs [range, 0–35/10 HPFs], *P* < 0.001), mean Ki-67 LI (mean, 1.15% ± 1.02% [range, 0%–5.3%] versus 4.06% ± 7.87% [range, 0%–35%], *P* = 0.002), and mean PHH3 MI (1.37 ± 1.37/10 HPFs [range, 0–6/10 HPFs] versus 2.77 ± 5.42/10 HPFs [range, 0–25/10 HPFs], *P* = 0.014) were all significantly lower in rectal than in nonrectal NETs (Figures 2(b)–2(d)).

### 3.3. Comparisons between Original WHO Grades and Grades Modified by PHH3

Classification of the 141 NETs according to the WHO grading system showed that 110 (78.0%) were of grade 1, 29 (20.6%) were of grade 2, and two (1.4%) were of grade 3.

To determine the PHH3 MI cutoff values that mostly closely matched the established WHO grade, we applied PHH3 MI in two ways (Table 2): (1) counting PHH3 MI according to the mitosis count on H&E slides, following by application of PHH3 MI to the WHO grading system instead of mitosis; (2) using a 4 PHH3 MI cutoff value, followed by application of PHH3 MI to the WHO grading system instead of mitosis or Ki-67 LI. Then, we generated a ROC curve to validate the optimal cutoff value, which showed an area under curve of 0.701 (95% confidence interval, 0.561–0.826), which was statistically significant (*P* = 0.007) (Figure 2(e)). At an optimal cutoff of 4, the sensitivity and specificity using 4 PHH3 MI to differentiate the WHO grade 1 and grades 2-3 were 73.3% and 31%, respectively.

Replacement of mitotic counts with the PHH3 MI in the WHO grading system resulted in 86 (61.0%) tumors being classified as grade 1, 53 (37.6%) as grade 2, and two (1.4%) as grade 3. The concordance rate of this modified system with the WHO grades was 75.9%. Replacement of mitotic counts with the PHH3 MI resulted in a change of grade of 36 tumors (25.5%), with 30 (21.3%) changed from grade 1 to grade 2 and six (4.3%) changed from grade 2 to grade 1. The association between these modified grades and the WHO grades was moderate (*κ* = 0.428) but statistically significant (*P* < 0.001).

The application of a PHH3 MI cutoff ≥4 in the WHO grading system resulted in 104 (73.8%) tumors being classified as grade 1 and 35 (24.8%) as grade 2. Use of this modified grading system with PHH3 MI ≥4 resulted in change of grade of 10 (7.1%) tumors, with eight (5.7%) changed from grade 1 to grade 2 and two (1.4%) changed from grade 2 to grade 1. The concordance rate of these modified grades with the original WHO grades was 92.9%, with almost perfect agreement between the two (*κ* = 0.810), a result that was statistically significant (*P* < 0.001).

Use of PHH3 ≥4 combined with the WHO grading criteria resulted in 10 tumors being reclassified (Table 3), nine rectal NETs and one gastric NET. Eight of these 10 tumors were upgraded by the addition of PHH3 MI to the WHO grading system compared with mitotic counts by the WHO grading system alone.

### 3.4. Prognostic Significance of the Inclusion of the PHH3 Cutoff

**Figure 3:**
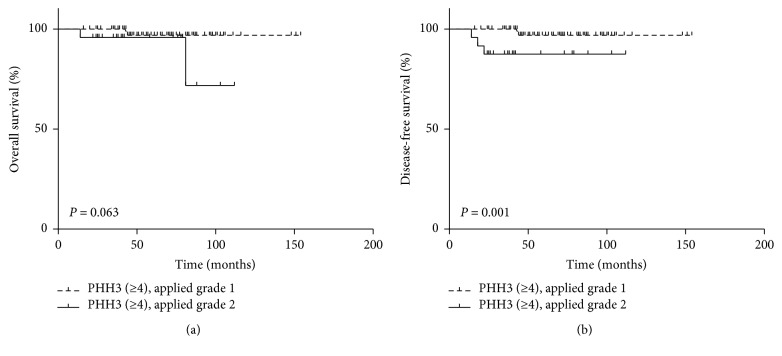
Impact of using PHH3 ≥4 combined with WHO grading criteria on overall survival and recurrence-free survival in patients with rectal NETs. Associations of PHH3 MI with (a) disease-free survival and (b) overall survival in patients with rectal NETs.

Because the use of PHH3 ≥4 in the WHO grading criteria yielded grades closest to those determined by the original WHO grading system, we analyzed the prognostic relevance of the combined criteria for overall survival and disease-free survival in patients with rectal NET (Figures 3(a)-3(b)). The modified grading system showed that disease-free survival was significantly worse (96.49 ± 7.10 months versus 150.81 ± 2.22 months; *P* = 0.001) and overall survival tended to be worse (*P* = 0.063), in patients with G2 than G1 rectal NETs.

## 4. Discussion

This study was designed to explore the diagnostic utility of PHH3 MI as an ancillary mitotic marker and the clinically relevant cutoff value of PHH3 MI in patients with gastrointestinal well-differentiated NETs, by comparing WHO grades and WHO grades modified by PHH3 MI. We found that a PHH3 MI cutoff of 4 was most similar to WHO grade.

The most accurate evaluation of mitoses in patients with NETs using the WHO grading system remains unclear, because mitoses may be mimicked by darkly stained or shrunken irregular nuclei, apoptotic bodies, and karyorrhectic debris, yielding false positives. In addition, diagnosis of mitoses is limited by the narrow cutoffs in mitotic counts between grades 1 and 2. PHH3 is only expressed during mitosis, not during interphase or apoptosis, making PHH3 a specific marker of mitosis [19, 20]. We found that mitotic counts correlated with both the Ki-67 LI and PHH3 MI, but its correlation with PHH3 MI was slightly higher, indicating that PHH3 MI is more closely associated with mitosis in gastrointestinal NETs. PHH3 only stains cells during the late G2 and M phases of mitosis [20], whereas Ki-67 is expressed throughout the cell cycle except in the G0 phase [26]. PHH3 would therefore stain far fewer tumor cells than Ki-67, resulting in a lower PHH3 MI.

Most determinations of the prognostic impact of mitoses in gastrointestinal NETs are based on the evaluation of mitoses by H&E staining [21]. Although the results using PHH3 correlated with mitosis on H&E slides [16, 27], it is unclear if these two types of mitoses have the same prognostic impact. In addition, no standards have yet been developed for the quantification in gastrointestinal NETs. PHH3 MI is comparable to the current WHO grading system but is superior to H&E and Ki-67, in predicting disease-free survival, with PHH3 appearing to be both easier to interpret and more accurate than current prognostic markers [14]. Evaluations in the present study of the prognostic utility of PHH3 MI instead of mitotic counts found that a PHH3 MI cutoff of 3 was no better than 3 mitotic counts per 10 HPFs in the WHO grading system for predicting outcomes in patients with rectal NETs. Of the 141 tumors, 36 showed discrepancies from the original WHO grades, with 30 upgraded and six downgraded when a PHH3 MI cutoff was used. Similarly, approximately one-third of discordant gastrointestinal stromal tumors were upgraded when determined by PHH3 application compared with H&E-stained slides [15]. The use of PHH3 in melanomas has been reported to upgrade 6–14% of tumors from pT1a to pT1b [16], indicating that replacement of mitotic counts by PHH3 MI in the grading system resulted in higher tumor grades. In contrast, a PHH3 MI cutoff of 4 could significantly distinguish between grades 1 and 2. Using this criterion, only 10 tumors showed discrepancies, with eight being upgraded and two (1.4%) downgraded. Furthermore, use of a PHH3 MI cutoff ≥4 in the WHO grading criteria instead of mitosis or KI-67 LI showed almost perfect agreement with the original WHO grades (*κ* = 0.810). Therefore, PHH3 MI ≥4 is likely to yield results comparable to the original WHO grades.

Use of a PHH3 MI cutoff ≥4 was associated with disease-free survival in patients with rectal NETs and could distinguish between grade 1 and grade 2 tumors. In contrast, this cutoff value was marginally significant in predicting overall survival in patients with rectal NETs. Thus, a PHH3 ≥4 cutoff value could approximate the results of the original WHO grading system in rectal NETs, as well as their prognostic correlations. Similarly, findings in pancreatic well-differentiated NETs, histologic grade, determined that ≥4 PHH3-stained mitoses/10 HPFs significantly correlated with patient survival [25].

Many studies in American and European populations [1–4] have shown that the majority of gastrointestinal NETs are located in the rectum, followed by the small intestine, colon, stomach, and appendix, and that the incidence of these tumors at all primary sites, especially the rectum and small intestine, increases with age [28]. In the present study, 115 (81.6%) of the 141 gastrointestinal NETs were located in the rectum, whereas only 26 (18.4%) were nonrectal NETs. Compared with nonrectal NETs, rectal NETs were associated with younger age, smaller tumor size, more superficial invasion, lower stage, lower grade, lower recurrence rate, and lower mortality rate. Most (83.5%) rectal NETs were classified as grade 1, whereas 41.3% of nonrectal NETs were of grade 2 or 3. Similarly, the primary tumor site distribution in our study was similar to that previously reported in the Korean, Japanese, and Chinese populations [7, 29, 30]. These findings suggest that the distribution of primary sites of gastrointestinal NETs may differ in Asian and Caucasian populations [7, 30].

In conclusion, the cutoff value of PHH3 ≥4 yielded results most similar to the original WHO grades. These findings suggest that this PHH3 MI cutoff may be a helpful adjunct prognostic strategy most likely reflecting the original WHO grades of gastrointestinal NETs. Although the number of patients in this study was relatively small, limiting the robustness of our conclusions, PHH3 appears to impart a useful ancillary marker for tumor grading. Additional studies are needed to confirm the optimal cutoff value of PHH3 MI for tumor grading of gastrointestinal NETs.

## Figures and Tables

**Table 1:** Associations of the clinicopathological characteristics of rectal and other gastrointestinal neuroendocrine tumors.

	Rectal NET	Nonrectal NET	*P*
	*n* = 115 (%)	*n* = 26 (%)	
Sex			0.148
M	75 (65.2)	13 (50.0)	
F	40 (34.8)	13 (50.0)	
Age (y)			0.001^*∗*^
<60	98 (85.2)	15 (57.7)	
≥60	17 (14.8)	11 (42.3)	
Tumor size (cm)			<0.001^*∗*^
0.1–1	111 (96.5)	20 (76.9)	
>1	4 (3.5)	6 (23.1)	
Tumor depth			0.001^*∗*^
T1	114 (99.1)	21 (80.8)	
T2-3	1 (0.9)	5 (19.2)	
LN metastasis			0.460
N0	113 (98.3)	25 (96.2)	
N1	2 (1.7)	1 (3.8)	
Distant metastasis			0.184
M0	115 (100)	25 (96.2)	
M1	0 (0.0)	1 (3.8)	
Stage			0.007^*∗*^
I	112 (97.4)	22 (84.6)	
II-III	3 (2.6)	4 (15.4)	
Grade			0.001^*∗*^
G1	96 (83.5)	15 (57.7)	
G2	19 (16.5)	9 (34.6)	
G3	0 (0.0)	2 (7.7)	
Mitosis/10 HPF	0.55 ± 0.79	2.62 ± 7.03	<0.001^*∗*^
<2	100 (87.0)	17 (65.4)	0.004^*∗*^
2–20	15 (13.0)	8 (30.8)	
>20	0 (0.0)	1 (3.8)	
Ki-67 LI (%)	1.15 ± 1.02	4.06 ± 7.87	0.002^*∗*^
<3	109 (94.8)	20 (76.9)	0.001^*∗*^
3–20	6 (5.2)	4 (15.4)	
>20	0 (0.0)	2 (7.7)	
PHH3 MI/10 HPF	1.37 ± 1.37	2.77 ± 5.42	0.014^*∗*^
<2	75 (65.2)	16 (61.6)	0.485
2–20	40 (34.8)	9 (34.6)	
>20	0 (0.0)	1 (3.8)	
Vascular invasion			0.375
Positive	22 (19.1)	7 (26.9)	
Negative	93 (80.9)	19 (73.1)	
Lymphatic invasion			0.363
Positive	18 (15.7)	6 (23.1)	
Negative	97 (84.3)	20 (76.9)	
Perineural invasion			0.123
Positive	12 (10.4)	0 (0.0)	
Negative	103 (89.6)	26 (100)	
Resection margin			1.000
R0	97 (84.3)	22 (84.6)	
R1	18 (15.7)	4 (15.4)	
Recurrence			0.001^*∗*^
Yes	6 (5.2)	7 (26.9)	
No	109 (94.8)	19 (73.1)	
Died			0.018^*∗*^
Yes	4 (3.5)	4 (15.4)	
No	111 (96.5)	22 (84.6)	

NET, neuroendocrine tumor; HPF, high-power field; LI, labeling index; MI, mitotic index. ^*∗*^Statistically significant. *P* value <0.05.

**Table 2:** Comparison of histologic grades combined with PHH3 staining and cutoff value of ≥4/10 HPFs.

	Total *N* = 141 (%)	WHO grade			*P*	*Kappa*
		Grade 1	Grade 2	Grade 3		
		*n* = 110 (%)	*n* = 29 (%)	*n* = 2 (%)		
Grades in replacement of H&E-mitosis by PHH3					<0.001^*∗*^	0.428
Grade 1	86 (61.0)	80 (80.0)	6 (20.7)	0 (0.0)		
Grade 2	53 (37.6)	30 (30.0)	23 (79.3)	0 (0.0)		
Grade 3	2 (1.4)	0 (0.0)	0 (0.0)	2 (100)		
Grades with PHH3 cutoff ≥4/10 HPFs					<0.001^*∗*^	0.810
Grade 1	104 (73.8)	102 (92.7)	2 (6.9)	0 (0.0)		
Grade 2	35 (24.8)	8 (7.3)	27 (93.1)	0 (0.0)		
Grade 3	2 (1.4)	0 (0.0)	0 (0.0)	2 (100)		

HPF, high-power field. ^*∗*^Statistically significant. *P* value <0.05.

**Table 3:** Comparison of clinicopathological features of tumors of grades stratified before and after use of PHH3 MI (≥4/10 HPFs) determining mitotic counts.

Patient number	Sex/age	Location	Size	Mitosis	Ki-67 LI	PHH3 MI	WHO grade	PHH3 grade
1	F/37	Rectum	0.5	0	0.7	4	1	2
2	M/47	Rectum	0.7	2	1.6	2	2	1
3	M/46	Rectum	0.5	2	2.8	1	2	1
4	M/49	Rectum	0.5	0	2.1	5	1	2
5	M/47	Rectum	0.5	0	2.5	6	1	2
6	F/35	Rectum	0.4	0	2	4	1	2
7	M/60	Rectum	1	0	2.4	4	1	2
8	F/55	Rectum	0.5	0	2.5	4	1	2
9	M/21	Rectum	0.5	0	2.5	6	1	2
10	F/56	Stomach	1	1	0.5	4	1	2

MI, mitotic index; HPF, high-power field; LI, labeling index.
